# Exploring synergistic patterns in bimanual distal limb movements through low dimensional representations

**DOI:** 10.1038/s41598-025-02680-x

**Published:** 2025-05-23

**Authors:** Prajwal Shenoy, S. K. M. Varadhan

**Affiliations:** 1 Department of Mechatronics, Manipal Institute of Technology, Manipal Academy of Higher Education (MAHE), Manipal, Karnataka 576104 India; 2 Department of Applied Mechanics and Biomedical Engineering, Indian Institute of Technology Madras, Chennai, Tamil Nadu 600036 India

**Keywords:** Kinematics, Synergies, Inertial measurement units, Bimanual, Biomedical engineering, Motor control

## Abstract

The human hand is a complex manipulator with many joints that can perform various tasks. Neuroscience research has demonstrated that to perform any posture, the brain does not control the individual joints but relies on coactivation patterns called synergies that simultaneously control a set of joints. A combination of these synergies can then be used to reconstruct a variety of postures. While such a hypothesis has been demonstrated for single-handed tasks, a question that is not well-explored is whether such synergies can simultaneously control the joints of both hands during bimanual tasks. This paper attempted to address this question by exploring synergies obtained by performing Principal Component Analysis (PCA) on the kinematic data recorded from both the dominant and non-dominant hands of the participants as they performed bimanual tasks. The ability of synergies to reconstruct postures from a lower-dimensional subspace was presented, and an analysis of the separability of postures was performed using a classification algorithm. The results showed that the first 3 synergies explained greater than 80% variance in data, indicating that a few bimanual synergies can be utilized to control the fingers of both hands. The first three synergies could reconstruct postures with a Root Mean Square Error (RMSE) of 4° and classify tasks with an accuracy of 90%, demonstrating that the task-related information was retained in the lower dimensional subspace. This could significantly reduce control complexities while designing robotic or prosthetic distal upper limb devices.

**Supplementary Information:**

The online version contains supplementary material available at 10.1038/s41598-025-02680-x.

## Introduction

The human hand possesses a complicated and intricate structure that provides remarkable agility, allowing it to perform manipulations easily and grasp various objects. The Central Nervous System (CNS) performs a sophisticated control of the hand involving a coordinated activity between multiple brain regions, resulting in a rich interplay between the muscles, tendons, and joints, thus resulting in the execution of a wide range of movements. How the CNS easily performs such a complex task has intrigued roboticists and neuroscientists alike in the last decade. The human hand is controlled via corticospinal projections from the brain, which include both monosynaptic connections to α motor neurons and polysynaptic pathways involving spinal interneurons. This organization is essential for voluntary and fine movement control^1–3^. The monosynaptic projections of the hand allow for rapid communication between the brain and the hand muscles, enabling us to perform dexterous tasks like writing, typing, and manipulating objects. On the other hand, due to the presence of monosynaptic projections, the strategy developed by the CNS can be explored by simply analyzing the movements (kinematics) and forces (kinetics) exerted by the fingers. Such strategies could help develop better robotic devices for grasping or developing hand prostheses^4,5^. Previously, researchers have hypothesized that, instead of controlling the individual joints of the fingers, the CNS relies on coactivation patterns called synergies to control the joints of the hand^6–10^. These synergies also referred to as strategies employed by the CNS, are learned representations in the brain; a weighted combination can result in any desired hand posture. To obtain these synergies, dimensionality reduction technique like PCA is applied to hand movement kinematics like velocities^11,12^ or joint angles^9,13,14^ measured using appropriate measurement systems like data gloves^6,10,12,15,16^, optical trackers^7–9^, electromagnetic trackers^17^, or Inertial Measurement Units (IMUs)^18^. Previous studies have demonstrated that the first 3 Principal Components (PCs-also called synergies) explain 80% of the variance in data, indicating that the CNS relies on a set of sparse synergies for controlling the complex hand^6,8,10^. Thus, any posture p can be computed using a weighted sum of top synergies S as represented in Eq. (1).

1 $$p = S \times z + p_{m}$$

here p is a column vector consisting of joint angles (ex: [21 × 1], where 21 is the number of joint angles), S is the synergy matrix of size $m \times n,$ where m represents the joint angles, and n represents the top n synergies. If the top 3 synergies are considered, S will have a size of [21 × 3]. The synergy matrix was obtained by performing PCA on the original zero-centered data matrix X with size [i × 21], where i is the number of rows. z is a control matrix with variables that can be optimized to produce any posture p upon multiplication with S. The values in matrix z can be thought of as adjustable knobs, which, when set to appropriate values, can produce the required posture. If the top 3 synergies are considered, z will have a size of [3 × 1]. Finally, $p_{m}$ is the column vector of mean joint angles. This is necessary since PCA is performed on zero-centered data after subtracting the mean from each column. Practically, the values in z could be from any measured physiological signals like EMG or EEG that could then be used to control the prosthetic hand with a certain number of joints.

Many studies have been performed in the domain of synergy analysis aimed at increasing our understanding of the intricate movement control strategies of the brain. Some of them include the exploration of time-varying synergies^19^, evaluation of dependency of synergies on task^8^, investigation of the use of synergies for enhancing learning^20^, the effect of the cutaneous impairment on synergies^9^, analysis of higher-order synergies^21^, the similarity of synergies across participants^22^ and comparison of various techniques like linear and non-linear methods for dimensionality reduction^16^.

One less explored area is the possibility of simultaneous control (hereafter called bimanual control) of both dominant (DO) and non-dominant (NDO) hands using bimanual synergies. Studies in neuroscience have demonstrated that the distal segments, like fingers, have a greater lateralized control with specific neurons in the right hemisphere dedicated to controlling the left hand (NDO) and vice versa^23,24^. The lateralization decreases, and the control becomes bilateral towards the proximal segments like shoulders and elbows, thus involving contribution from both hemispheres^25^. In other words, considering the matrix z in Eq. (1), this indicates that separate knobs (some representing the right hemisphere and some representing the left) are required to control the distal DO and NDO hands. In contrast, a single knob (that represents control from both hemispheres) that controls the bimanual synergies (involving joints of both hands) is sufficient for the control of proximal segments. Thus, distal segments require separate focused control compared to the proximal segments. However, one preliminary study^14^ demonstrated that even distal DO and NDO segments could be controlled easily using single knobs through bimanual synergies.

While further studies have shown success in bimanual control of proximal segments^11^, studies showing the possibility of bimanual control for distal segments are scarce. Despite deeper lateralization, the possibility of bimanual control of distal segments could be answered through task demands. Previous studies have shown that when task demands are lower or tasks are familiar, even distal segments exhibit bimanual control involving both hemispheres and subcortical regions^26–28^. In contrast, as task demands increase, the control becomes focused, requiring the involvement of the contralateral hemispheres of the brain. Hence, we hypothesize that, for tasks involving activities of daily living that are well-practiced, the activities as dictated by the bimanual synergies are mainly processed at the subcortical level independently from the unimanual-bimanual strategies. Such synergies could greatly simplify the control architecture of bimanual robotic hands, thus cutting down on actuator and control costs and space requirements.

Thus, in this paper, based on the preliminary study^14^, we attempt to derive bimanual synergies from kinematic data obtained from participants as they perform different bimanual tasks. An IMU-based kinematic measurement system will be utilized for this. The description of the obtained synergies and the ability to reconstruct postures from sparse synergies will be analyzed. Finally, as an application, synergies will be used to reduce the dimension of the original data by projecting the data into the lower dimensional subspace. A classification algorithm based on logistic regression will be used to assess the separability of postures in lower-dimensional subspace. Greater separability in lower dimensions indicates that the postural information is retained in that space.

## Materials and methods

### Participants

Sixteen right-handed participants, eight males and eight females of age 29 ± 4.26 (mean ± S.D), participated in the study. The participants provided written informed consent and reported no history of neuromotor disorders and hand or arm injury. The IIT Madras Institutional Human Ethics Committee (IITMIHEC) (IEC/2022-02/SKM/01/07) approved the experimental procedures. All the experimental sessions were performed in accordance with the relevant guidelines and regulations approved by the Institute Human Ethics Committee of the Indian Institute of Technology Madras.

### Details of the data acquisition system

**Fig. 1:**
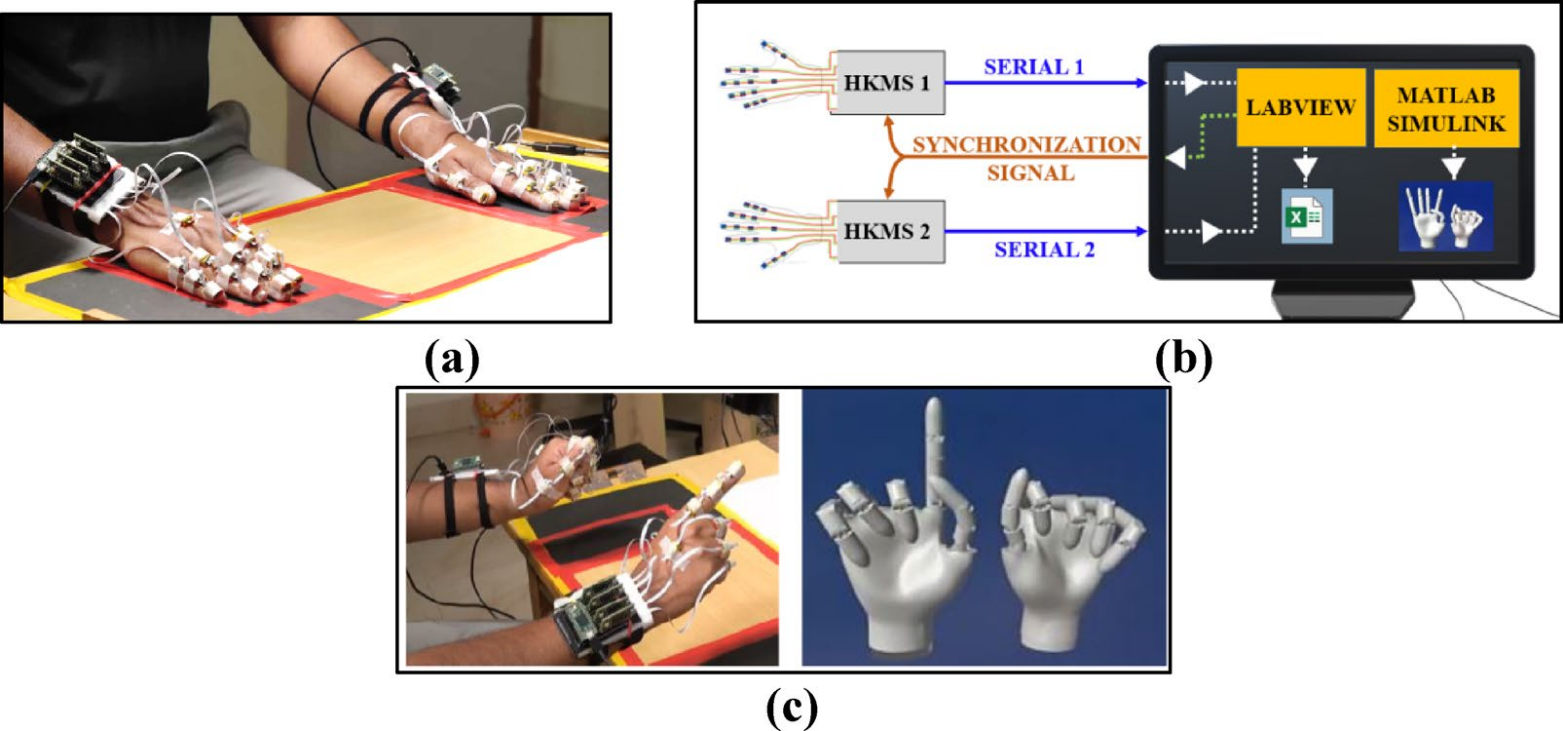
Details of the data acquisition process. (**a**) Sixteen sensors per hand are attached to the phalanges that record the kinematics of all joints. A microcontroller-based receiver system is used to collect data at 100 Hz. (**b**) A schematic of the data collection process. A LabVIEW custom program synchronizes the device on both hands and receives data from all the sensors of DO and NDO hands simultaneously. Image generated using Microsoft PowerPoint (Version 2503, https://www.microsoft.com/en-us/microsoft-365/powerpoint) (**c**) Snapshot from a real-time animation using MATLAB Simulink (Version R2024B, https://www.mathworks.com/products/simulink.html).

An Inertial Measurement Unit (IMU) sensor-based hand kinematic acquisition system (HKAS) was used for the study. Each IMU consists of an accelerometer, gyroscope, and magnetometer and fuses the data to obtain the three-dimensional orientation of an object in space using an appropriate filter. The device’s description, design, and validation have been previously presented^18^. The kinematic acquisition system consists of 2 HKAS devices, one for each hand. Briefly, each HKAS consists of 16 sensors that are placed on the 15 phalanges of the fingers (proximal, middle, and distal) and one on the wrist. These sensors measure the orientation of 15 joints of the hand (Metacarpophalangeal joint (MCP), proximal Interphalangeal joint (PIP), and distal phalangeal joint (DIP) for each finger. For the thumb, the sensors measure the orientation of the carpometacarpal joint (CMC), metacarpophalangeal joint (MCP), and interphalangeal joint (IP). Thus, for both hands, a total of 32 sensors are required to measure the orientation of the joints. An image with sensors attached to a participant’s hands is shown in Fig. 1a. These sensors transmit data to a receiving device consisting of a series of microcontrollers in a master–slave arrangement that transmits the data to the PC via serial communication at 100 Hz sampling frequency. The device has a static accuracy of 2° and a dynamic accuracy of 4°^18^. The 4° accuracy is considering very fast and large number of repetitive movements. The accuracy reduction is due to the drift that occurs when fast repetitive movements are performed (usually greater than 3 repetitive movements). In the current study, the tasks were set to ensure that no more than 3 repetitive movements were performed. Repetitive movements were given a pause in between wherever required. Hence the dynamic accuracy would be better. The following steps are followed to receive data from two HKAS devices simultaneously in real time. First, a synchronization signal through Serial communication is sent to the HKAS devices through LabVIEW to ensure the devices are synced (See Fig. 1b). Upon receiving the sync request, both HKAS devices start transmitting quaternion data from all sensors using two serial lines. For every timestep, each device outputs 64 values (16 sensors × 4 valued quaternion). Upon receiving the data in LABVIEW, for every timestep, the data from two devices is concatenated into a single line data comprising 128 values (16 sensors × 4 valued quaternion × 2 HKAS devices). The data is collected for every trial at 100 Hz and is saved in an Excel file for further analysis. This is demonstrated in Fig. 1b. A hand model was built using SOLIDWORKS and imported to MATLAB SIMULINK to animate the hand movements. Still images from a video measuring bimanual kinematics are shown in Fig. 1c. The animation was used only to check the device’s correctness and for posture visualization. The movement feedback was not provided to the participants.

**Fig. 2:**
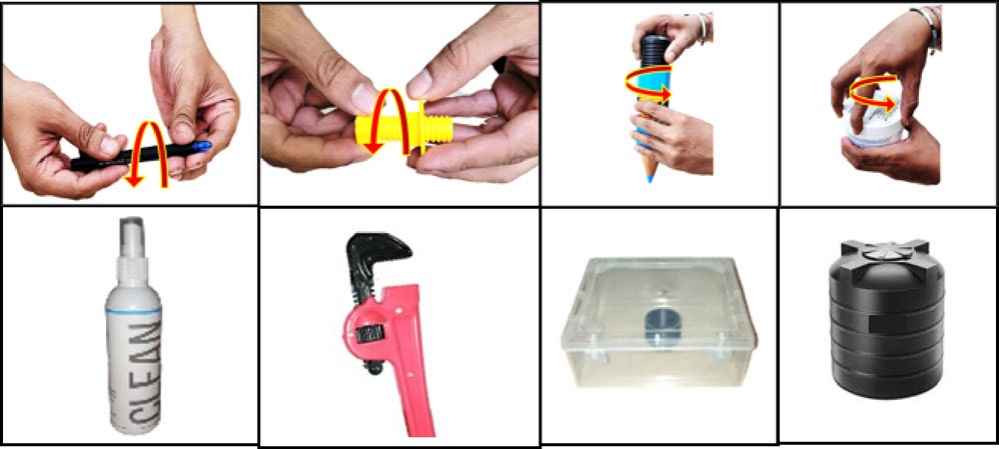
Tasks performed in the experiment: Rotation of objects requiring very small, small, large, and big apertures using the pen, screw, bottle, and gel box, respectively. Other tasks involved opening the lid of a bottle and unscrewing the top, manipulating the pipe wrench, placing and removing objects from a box, and inserting coins into a piggy bank. All images are photos of actual objects used in the study.

### Experimental protocol

The participants had to manipulate objects with the DO hand while the NDO hand held the object steady. There were 8 tasks in which participants were required to manipulate 8 objects (See Fig. 2). Manipulation of each object was repeated 10 times. The duration for each trial was 8 seconds. The first four tasks involved rotation tasks using objects of varying sizes. These tasks had manipulation apertures varying from small to medium to large. This was done using the pen, screw and nut, pencil box, and gel bottle. For all these 4 tasks, the participant had to pick the object from the resting position using the NDO hand in set 1 and the DO hand in set 2. The other hand was used to rotate the pen barrel, nut, box cap, and gel box lid for all 4 cases in the following fashion. 3 clockwise rotations followed three rotations in the counterclockwise direction. (This was done to ensure minimal drift errors in the sensor). After this, the object was kept back, and both hands were instructed to return to the original position. This movement was repeated ten times. The fifth task involved the following steps. The participants were required to hold the bottle with one hand after lifting it from the home position. In the first trial, the other hand was used to pull open the lid, place it on the table, and rotate the inner cap three times counterclockwise. The object was returned to the rest position, and the hand returned to the home position. In the second trial, the object was again lifted with one hand; the other hand was instructed to rotate the inner cap thrice in a clockwise direction, push-fit the cap, and place the bottle in the rest position, following which the hands were placed back in the home position. The procedure continued till the end of ten trials. In the sixth task, the pipe wrench was held using one hand, and the other hand was used to rotate the screw of the wrench using only the thumb and the index finger while the other fingers were flexed. The movements of the thumb and index finger were out of phase. i.e., when the index finger was flexed, the thumb was extended, and vice versa.

**Fig. 3:**
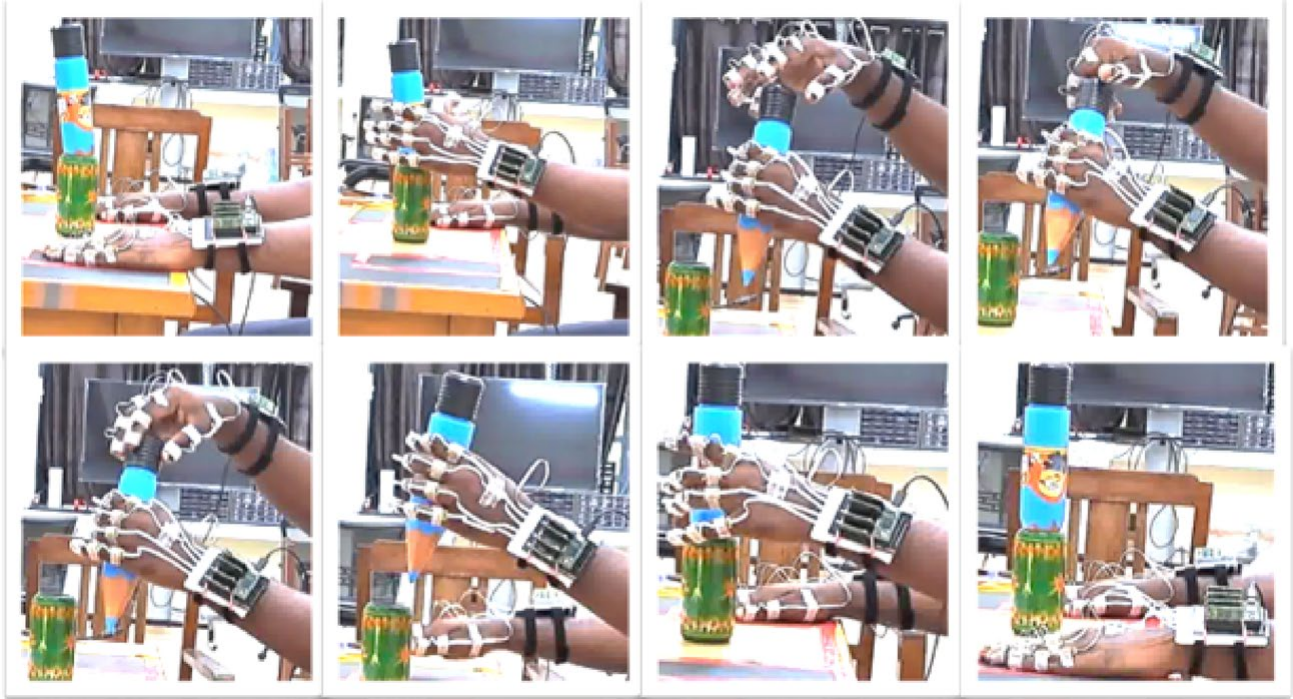
Still images of a video recorded during the experiment as a participant performed the bimanual task of opening and closing the lid of a cylindrical box. In 8 s, the participant lifted the object with the NDO hand and performed manipulation of the lid with the DO hand. A supporting object (a green wooden tumbler) was used to prevent the object from rolling when placed on the table. This makes sure that all the participants lift objects from the same position, thus ensuring consistency across participants.

At the end of the task, the object was kept back in position. In the seventh task, the participants were asked to lift the box with one hand and use the other to perform the following operation: 1. Open the lid, lift the object from the box, and place it on the table. Following this, the object was kept back in the resting position. In the second trial, the box was lifted with one hand, and the other hand was used to keep the object back in the box and close the lid. Upon completion, the object was returned to the table, and the hands rested. The procedure continued till the end of ten trials. In the last task, the piggy bank was held with one hand, and the other hand was used to pick coins and drop them in the piggy bank slot. The participants were required to pick three coins in each trial (to ensure minimal drift errors). The experimenter performed and demonstrated each task before the beginning of the experiment. Still images of a participant performing Task 3 in the first set are shown in Fig. 3. As can be seen from the figure, the DO hand is used to perform manipulative tasks (opening and closing the lid), and the NDO hand is used to hold the object steady. A detailed description of the tasks performed is provided in Table S1 in supplementary materials. After a break of 10 min, the same set of tasks were repeated with the roles of the hands reversed. The DO hand held the object steady, whereas the NDO hand was used to make manipulative movements. This data with roles of hand reversed is not analyzzed and presented in this paper.

### Data analysis

#### Computation of relative joint orientation

To compute the orientation of any joint, the quaternion data from the 2 adjacent sensors were multiplied using Eq. (2)

2 $$q_{1relative2} = q_{2}^{conj} \otimes q_{1}$$

Here $q_{2}$ and $q_{1}$ represent the orientation of the proximal and distal phalange, respectively. $\otimes$ represents quaternion multiplication, and conj represents quaternion conjugate operation.

#### Sensor calibration

The IMUs are required to be calibrated before the beginning of the experiments. A detailed description of the calibration procedure is described in^18^. The first calibration is for the gyroscope, wherein, to perform the calibration, the sensors must be kept as steady as possible. However, the system was switched ON in this experiment before attaching the sensors to the hand. In this way, the gyroscope would be calibrated immediately after switching ON. The participants didn’t need to hold their hands steady to calibrate the gyroscope. To calibrate the accelerometer, the participants had to orient their hands in steps of 45° for 3 or 4 steps before which the accelerometer was calibrated. The participants were asked to wave their hands to calibrate the magnetometer, imitating an infinity symbol. Thus all 3 calibration routines would be completed. The entire procedure could be completed in 20 s.

#### Sensor-to-segment alignment

For each trial, a sensor-to-segment alignment was achieved by averaging 50 samples (collected from 0.3 to 0.8 s, when the hand was at the home position) of the relative quaternions for each joint. This approach was feasible because each trial began after a one-second delay, during which the hand was held in a static, adducted home position. The averaged quaternion values were then used as a reference, with all relative quaternions in the trial expressed relative to these averaged values to achieve alignment. The sensors on the thumb, however, were excluded from this alignment process.

#### PCA to obtain bimanual synergies

**Fig. 4:**
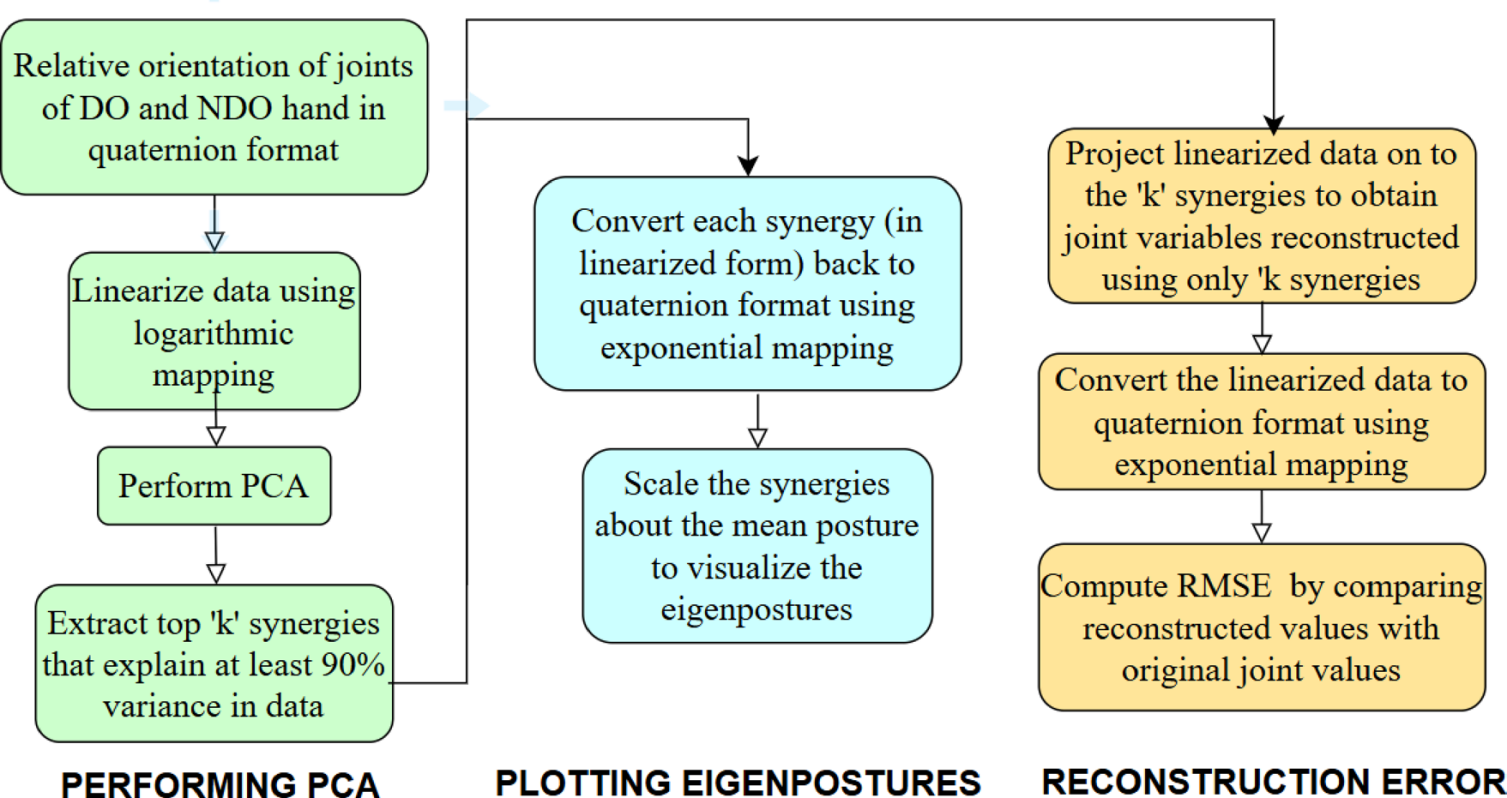
Flowchart depicting the analysis methodology. The original data is analyzed by visualizing the eigen postures, and reconstruction error. The procedure involves the conversion of quaternions to linear space and vice versa since quaternions are defined on a nonlinear manifold. The flowchart was created using an online flowchart maker, draw.io (https://app.diagrams.net/).

Quaternions obtained from the IMUs represent orientation in a nonlinear domain. Hence, a linear dimensionality reduction technique like PCA cannot be applied directly to the quaternion data matrix. For this purpose, the quaternions are linearized using logarithmic mapping, and PCA is applied to the linearized vectors. Conversion from the linear vectors to the quaternions is done using exponential mapping^29,30^. This method was first presented in^29^. This method has been applied previously in the dimensionality reduction of hand kinematics in^13,17,18^. PCA is computed for each participant separately, and the results are averaged across participants wherever applicable. A brief description of the synergy extraction procedure is presented and is also shown as a flowchart (Fig. 4).

- The data of a participant consists of the relative orientation of all joints of both the DO and NDO hands. The size of the data matrix is 64,000 (800 samples /trial × 10 trials × 8 tasks) × 120 (30 joints × 4 valued quaternions).
- This data is linearized since quaternions are defined in a nonlinear domain, and PCA is not applicable. Upon linearization, the 4-valued quaternion gets converted to a 3-valued vector. Hence, the size after linearization is 64,000 × 90
- A PCA is performed on the linearized data, and top ‘k’ synergies that explain at least 90% variance in data are selected.
- The extracted synergies are first visualized by plotting the eigenpostures. For this purpose, the extracted synergies (which are linearized) are converted back to quaternion format and plotted using a hand model in MATLAB. The synergies are scaled to visualize the movements encoded by each synergy.
- Secondly, the ability of the synergies to reconstruct postures from the reduced synergy space is tested. For this purpose, the linearized data is projected onto the reduced synergy space to be reconstructed. The reconstructed data is converted to quaternion format for comparison with the original data. The RMSE error is computed and presented.

A thorough description of the above-mentioned steps, along with the equations used, is provided in supplementary material “Principal Component Analysis to obtain bimanual synergies” Section.

#### Classification of postures in latent dimensional subspace

To classify postures in the latent space, the linearized data (64,000 × 90) was projected onto the top k synergies (90 × k) to obtain PC scores (64,000 × k). For instance, if the top 3 synergies were selected, the data size would be 64,000 × 3. For each trial in the task, 50 sample points (0.5 s) of data were selected between 3.5 and 4 s for comparison. Thus, the data size for classification for each participant was 4000 (50 samples × 10 trials × 8 tasks) x k. The data was split in the ratio of 60:40, where 60% of the data was used for training and 40% for testing. The classification was performed using logistic regression for each participant separately, and the classification accuracy was reported to quantify data separability in the latent dimensions.

## Results

### Analysis of bimanual synergies

**Fig. 5:**
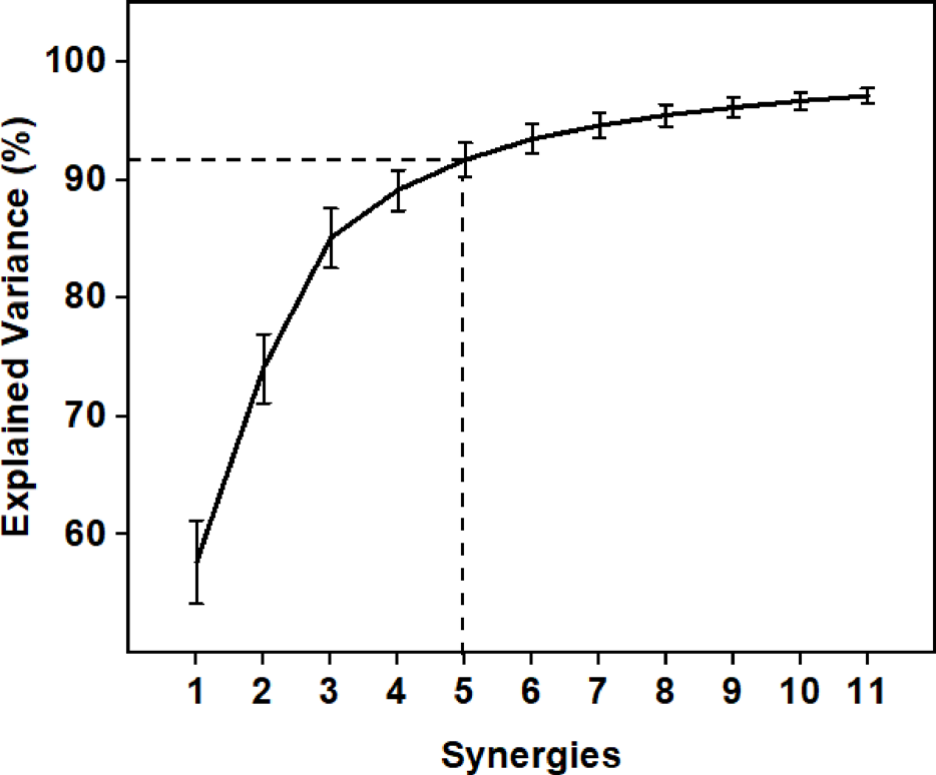
Plot of synergies versus explained variance computed using the cumulative eigenvalues obtained from the PCA analysis. Bars indicate Standard Deviation (S.D). The first five synergies account for greater than 90% variance in the data.

The eigenvalues obtained from the bimanual analysis of the DO and NDO hand movements are demonstrated through the scree plot in Fig. 5, which illustrates the variance (cumulative) explained by each synergy. The first synergy alone could explain around 55%variance in data. The second and third synergies, along with the first synergy accounted for greater than 70% and 80% variance in the data, respectively. This shows that lower-dimensional control using bimanual synergies efficiently controls the movement of the fingers of both the DO and NDO hands. The first 5 synergies that account for greater than 90% variance in the data are used for the rest of the analysis.

**Fig. 6:**
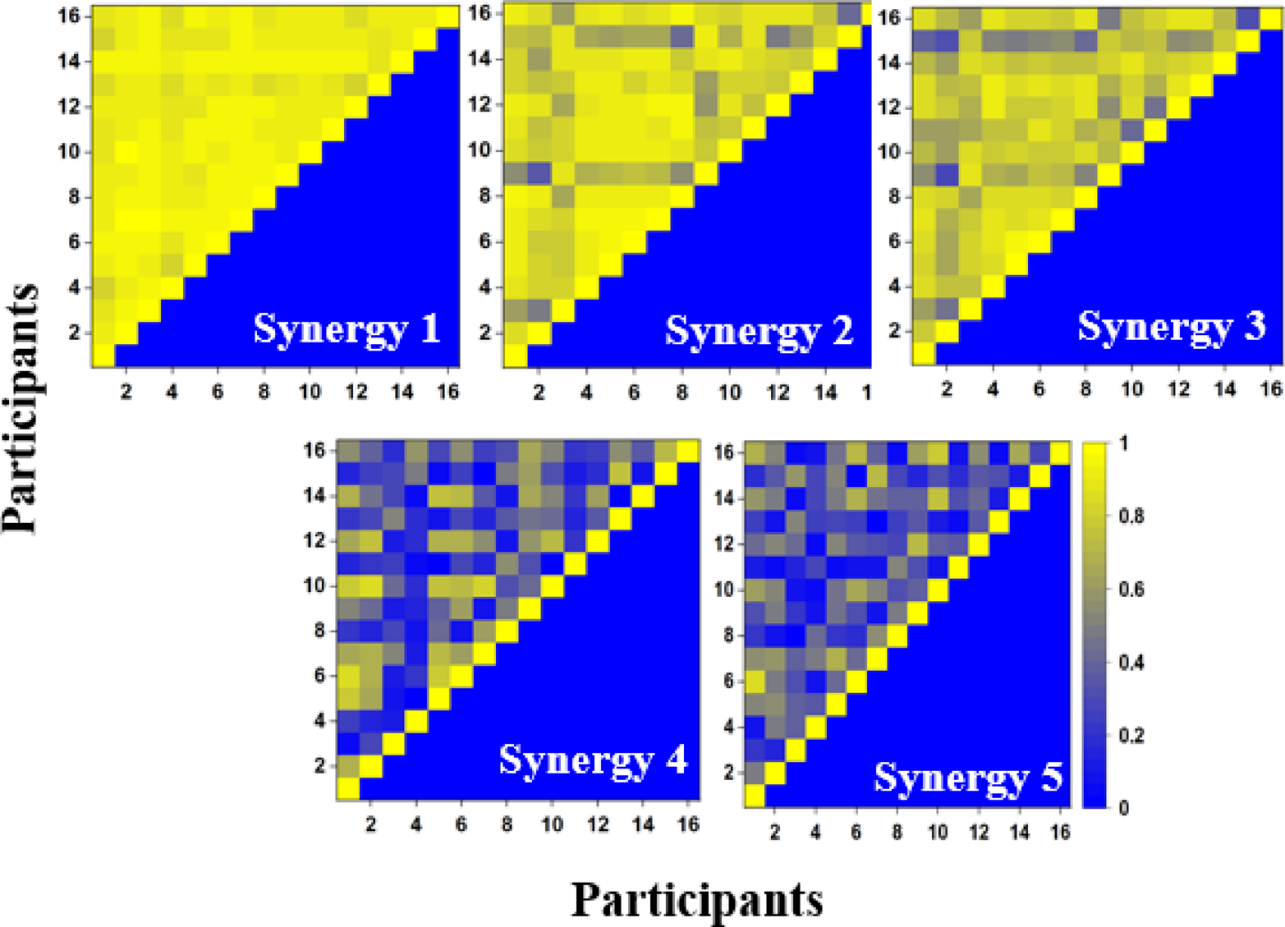
Pearson’s correlation coefficient computed between all possible pairs for a particular synergy of all participants. Each sub figures represent the correlation coefficient between (**a**) Synergy 1 (**b**) Synergy 2 (**c**) Synergy (**d**) Synergy 4 and (**e**) Synergy 5 of all participants. Absolute values of the correlation coefficient are plotted.

To check for similarity in synergies across participants, a Pearson’s correlation coefficient was computed for a given synergy across all participants. For example, the first synergy of all participants was grouped. A correlation coefficient was computed across all possible pairs. The results of this analysis are shown in Fig. 6. The first three synergies were similar across participants, whereas synergies 4 and 5 differed. Thus, the finer details of the postures encoded in the higher-order synergies differed across participants due to participant-specific strategies.

**Fig. 7:**
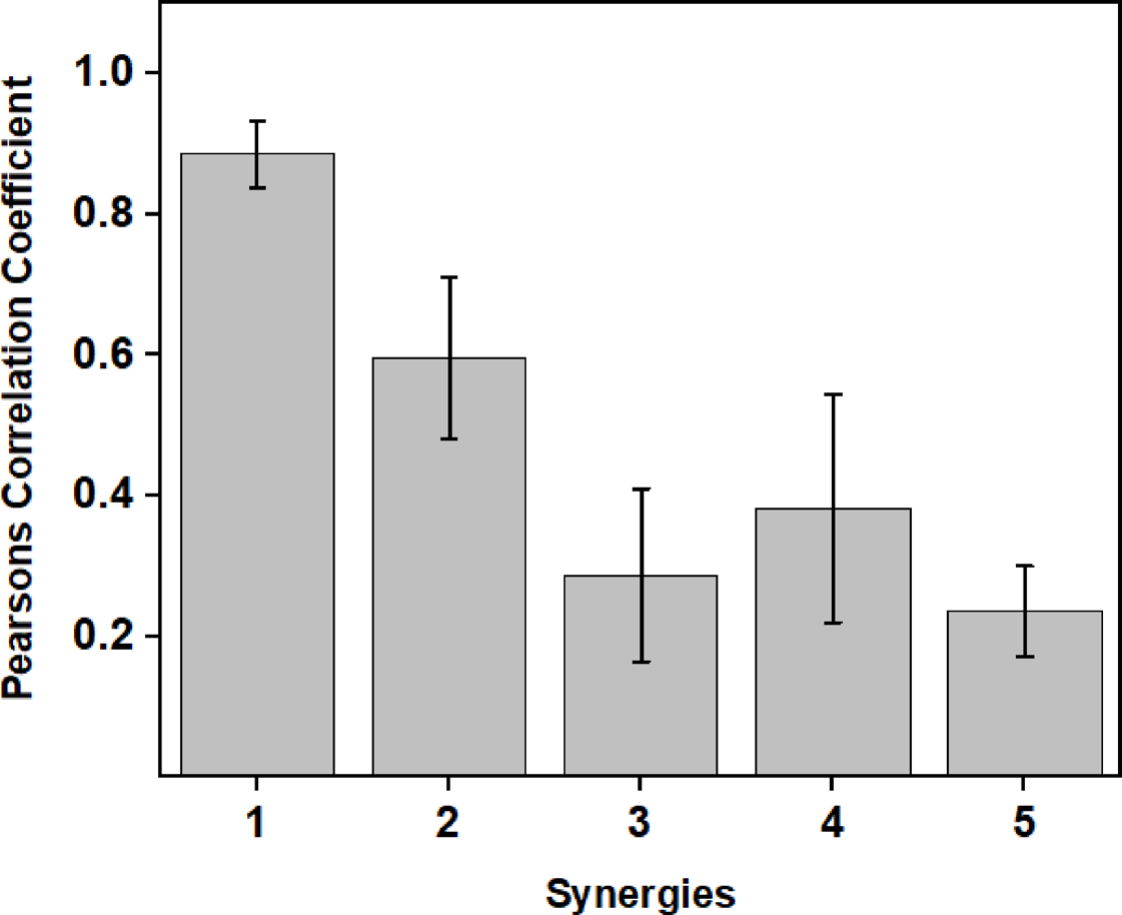
Pearson’s correlation coefficient between the DO and NDO hand for each synergy averaged across participants. The error bars indicate S.D. A higher correlation coefficient between the synergies of DO and NDO hands for the first synergy indicates symmetric movements.

To identify how the synergies encode movement for the DO and NDO hands, Pearson’s correlation coefficient was computed between the synergies of the DO and the NDO hand. The correlation coefficient was averaged across all participants and is presented in Fig. 7. From the figure, the first synergy shows a greater correlation between the DO and the NDO hand, whereas the correlation coefficient gradually decreases for higher-order synergies. This indicates that the symmetric movements are encoded in the lower-order synergies, whereas the asymmetries are encoded in the higher-order synergies. This is also in line with a previous bimanual study on arm movements^11^. However, in the current study, the movements had no symmetric manipulations. The symmetric movements in the first synergy could probably be due to brief symmetric movements that occur during the task. For example, in task 3, while the DO hand holds the pencil box, a similar posture is attained by the NDO hand at the instant it makes the first contact with the lid.

**Fig. 8:**
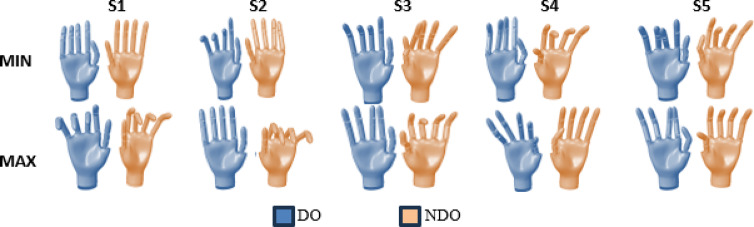
Variation of Eigen postures between the maximum and minimum limits for the first 5 synergies. The first synergy encodes symmetric movements between the DO and NDO hands. Higher-order synergies are asymmetric between the DO and NDO hands. The images are snapshots from the animation of a hand model simulated in Matlab Simulink (Version R2024B, https://www.mathworks.com/products/simulink.html).

The eigen postures are plotted for the first 5 synergies in Fig. 8 to visualize the bimanual synergies. As mentioned, the first synergy (S1) encodes symmetric movements between the DO and the NDO hands. This involves the PIP flexion of the index, middle ring, and the little fingers of both DO and NDO hands. The second synergy, S2, encodes movements of only the PIP joint of the middle, ring, and little fingers for the DO hand, whereas the synergy encodes movement of the MCP and PIP joint of all fingers for the NDO hand. This is probably because the NDO hand is involved in grasping a wide variety of objects that require the involvement of both PIP and the MCP joints. However, since the DO hand is involved in manipulative movements, most can be performed using a slight flexion of the PIP joints and some abduction/adduction movements of the MCP joints. The third synergy (S3) encodes only slight movements at the PIP joint for the DO hand, whereas the NDO hand mimics a grasp varying from lumbrical to cylindrical grasp. The fourth synergy (S4) mimics a pinch grasp (like that used while picking coins and dropping in a box) while NDO hand encodes a thumb-finger opposition grasp involving the PIP joints of all fingers and the IP joint of the thumb. The fifth synergy (S5) encodes abduction adduction movement of the finger MCP joints required for manipulation in the DO hand. The NDO hand encodes movement of the thumb IP joint.

**Fig. 9:**
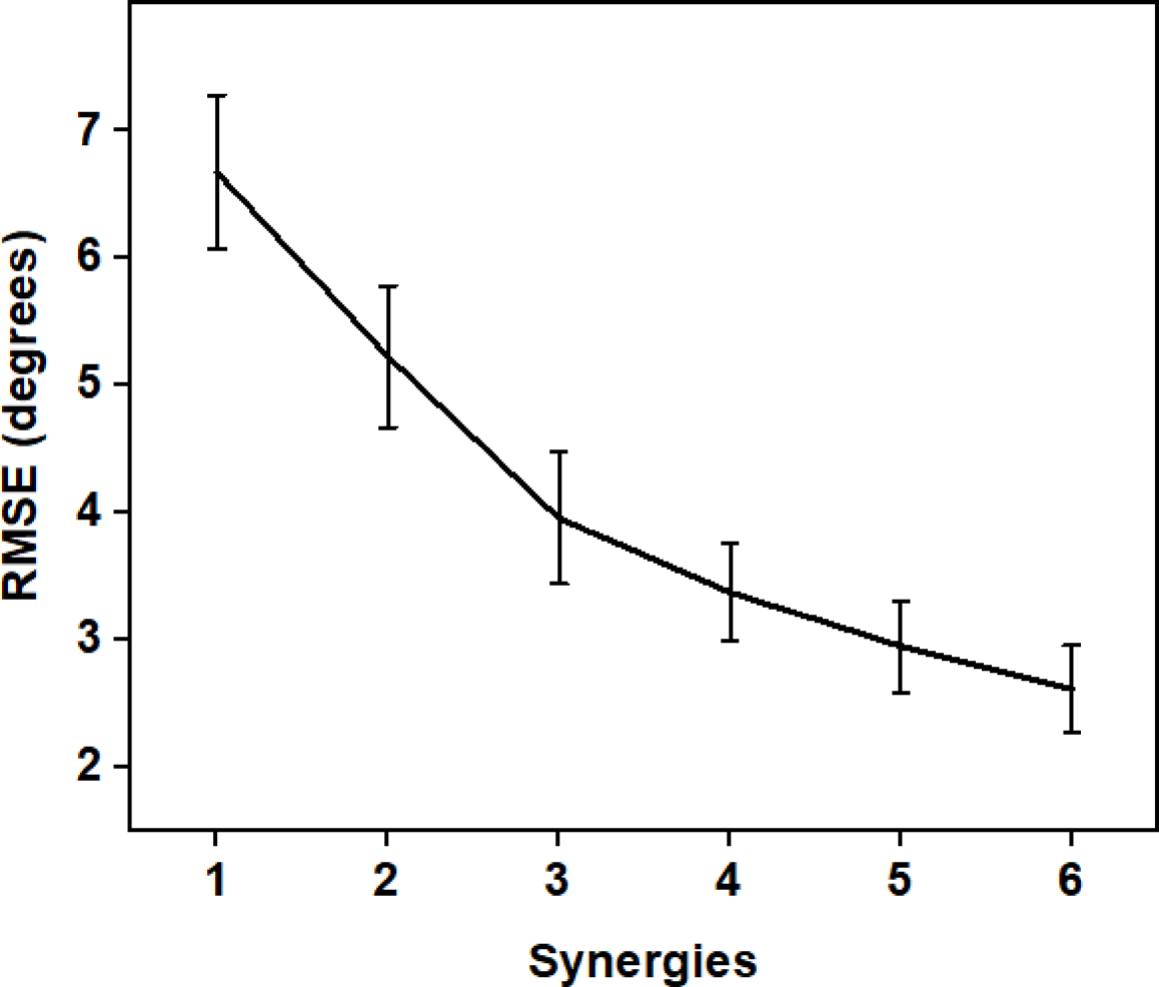
Variation of Eigen postures between the maximum and minimum limits for the first 5 synergies. The first synergy encodes symmetric movements between the DO and NDO hands. Higher-order synergies are asymmetric between the DO and NDO hands.

To investigate the ability of synergies to reconstruct postures from the latent space, the movements of all tasks are projected onto the lower dimensional synergy space and reconstructed. The reconstruction error (RMSE) averaged across all participants is shown in Fig. 9. Using the first synergy alone, the postures could be reconstructed to an accuracy of less than 7°. Upon adding more synergies, the reconstruction error reduces, and a reconstruction error of 4° is possible with just 4 synergies.

### Analysis of task reconstruction from sparse synergies

**Fig. 10:**
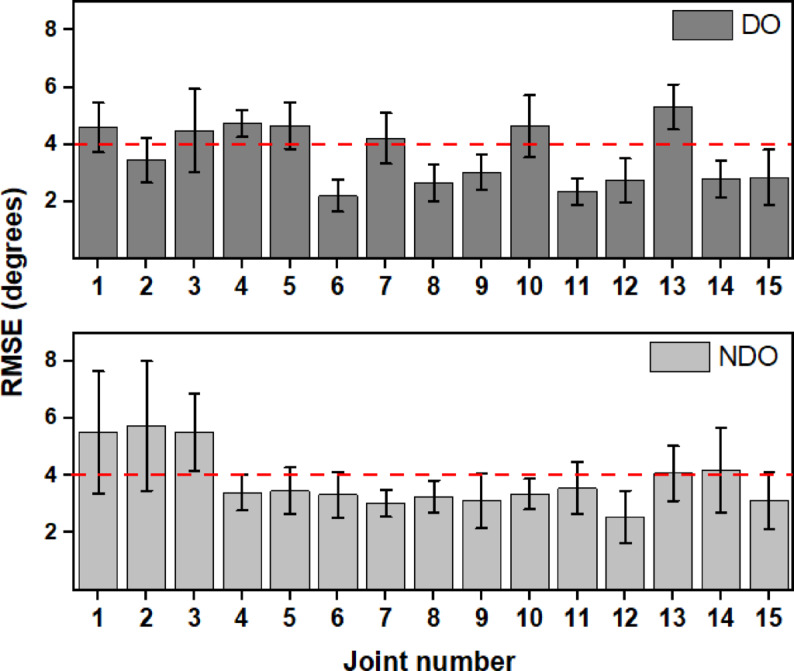
Reconstruction errors of individual joints computed using only 3 synergies for the DO hand (top) and NDO hand (bottom). Joints 1–3 indicate the CMC, MCP, and IP joint of the thumb. Joints 4–6 represent the MCP, PIP, and DIP joints of the index finger, joints 7–9 represent the MCP, PIP, and DIP joints of the middle finger, joints 10–12 represent the MCP, PIP, and DIP joints of the ring finger, joints 13–15 represent the MCP, PIP, and DIP joints of the little finger.

To analyze the reconstructed joint movements, the reconstruction errors for the first 3 synergies are plotted separately for each joint of the DO and NDO hands. The results are shown in Fig. 10. For the DO hands, the results show that the reconstruction errors are higher (> 4° RMSE) for the Thumb CMC, Thumb IP, Index MCP, and Index IP joints. The reconstruction errors are also higher for the middle, ring, and little finger MCP joints. In the case of the NDO hand, the thumb joints exhibit a greater error.

**Fig. 11:**
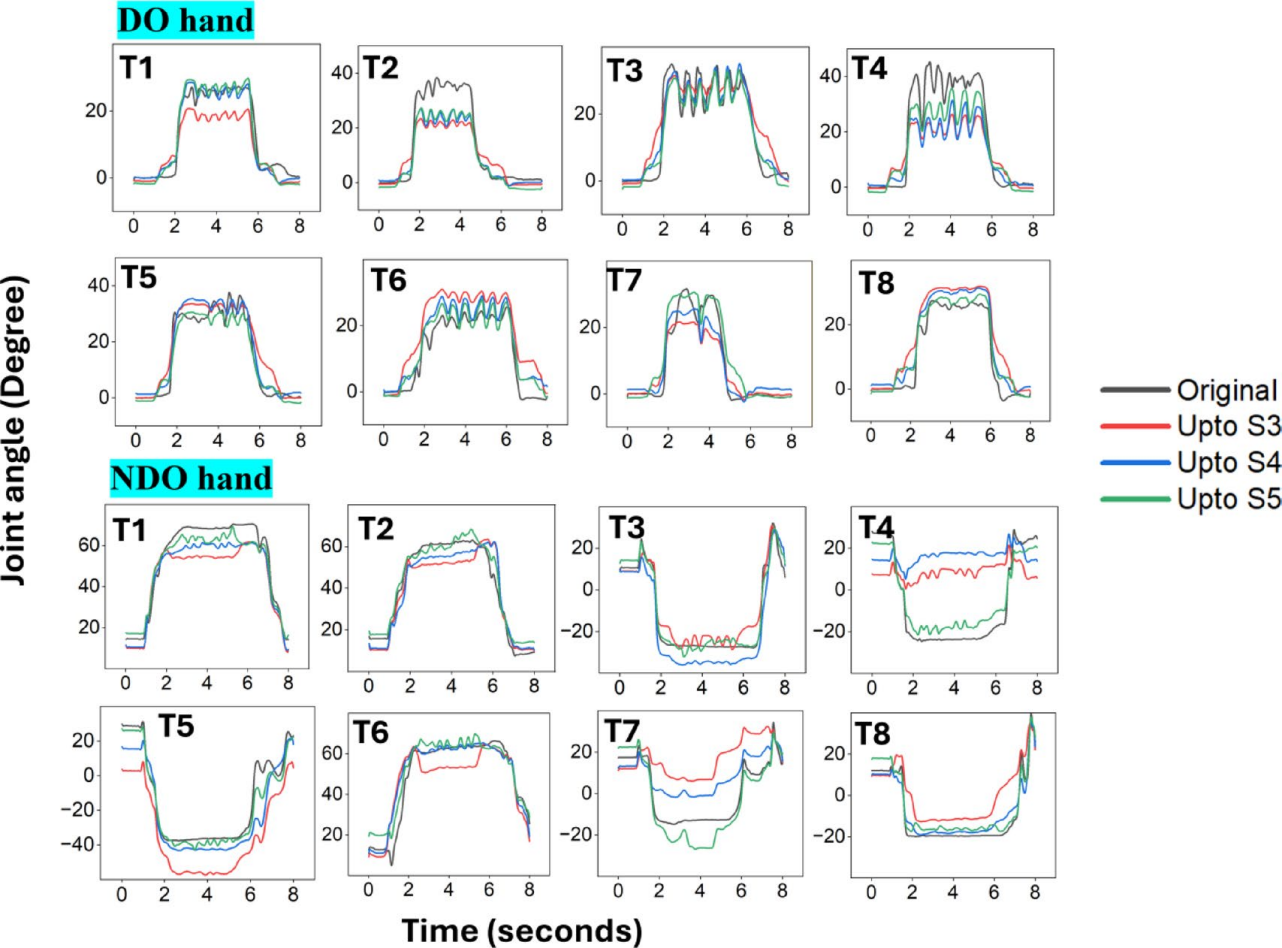
Joint angles reconstructed using the first 3–5 synergies are plotted. For the DO hand, the joint angles of the flexion extension of the little finger MCP joint of a participant (P2) that exhibited maximum RMSE (> 6°) was plotted for all tasks. On similar lines, for the NDO hand, the thumb IP joint of a participant (P11) who demonstrated maximum RMSE (> 12°) was plotted for all tasks.

For further visualization of individual joint movements, the flexion extension of the little finger MCP joint of a participant (P2) that exhibited maximum RMSE (> 6°) was plotted for all tasks. On similar lines, the thumb IP joint of a participant (P11) who demonstrated maximum RMSE (> 12°) was plotted for all tasks. The results are shown in Fig. 11. Additionally, the effect of adding more synergies (up to S5) is plotted to observe the improvement in reconstruction error upon adding higher-order synergies. The results indicate that, for the DO hand, the first three synergies reconstruct the joint movements for most tasks except for tasks T1, T2, and T4. Adding more synergies improves the reconstruction error for task T1. However, tasks T2 and T4 require the addition of higher-order synergies to improve reconstruction. Similarly, the first 3 synergies are sufficient for most tasks except tasks T4, T5, and T7. However, adding more synergies increases the reconstruction accuracy, and adding up to 5 synergies provides better reconstruction for all tasks. However, it should be noted that these results are for that participant who demonstrated the maximum reconstruction error. Additionally, it can be observed that for the NDO hand, the original postures attain a steady state after grasping the object. However, the reconstructed postures contain slight oscillating movements. These oscillations are inherited from the DO hand since the bimanual synergies were obtained using the DO and NDO hands. Additionally, it can be observed that the oscillations increase upon adding higher-order synergies.

**Fig. 12:**
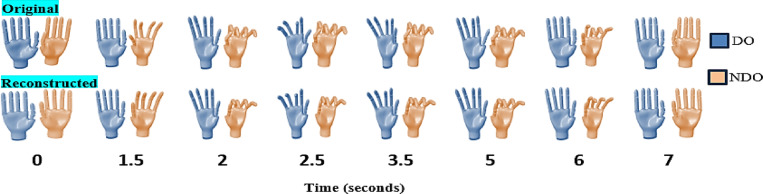
Visualization of Task 3 for a randomly selected participant. The postures reconstructed using only 3 synergies are compared with the temporal progression of original movements. The images are snapshots from the animation of a hand model simulated in Matlab Simulink (Version R2024B, https://www.mathworks.com/products/simulink.html).

The reconstructed postures of a randomly selected participant for task 3 are shown in Fig. 12. The posture was reconstructed using 3 synergies. Visually, the differences between the original and reconstructed postures are minimal. As discussed previously, errors in the flexion of the thumb IP joint can be seen between the original and reconstrued postures. Hence, for applications involving VR/AR, a few bimanual synergies could be used to reconstruct any postures with minimal observable differences.

### Classification of tasks in lower dimensional subspace

**Fig. 13:**
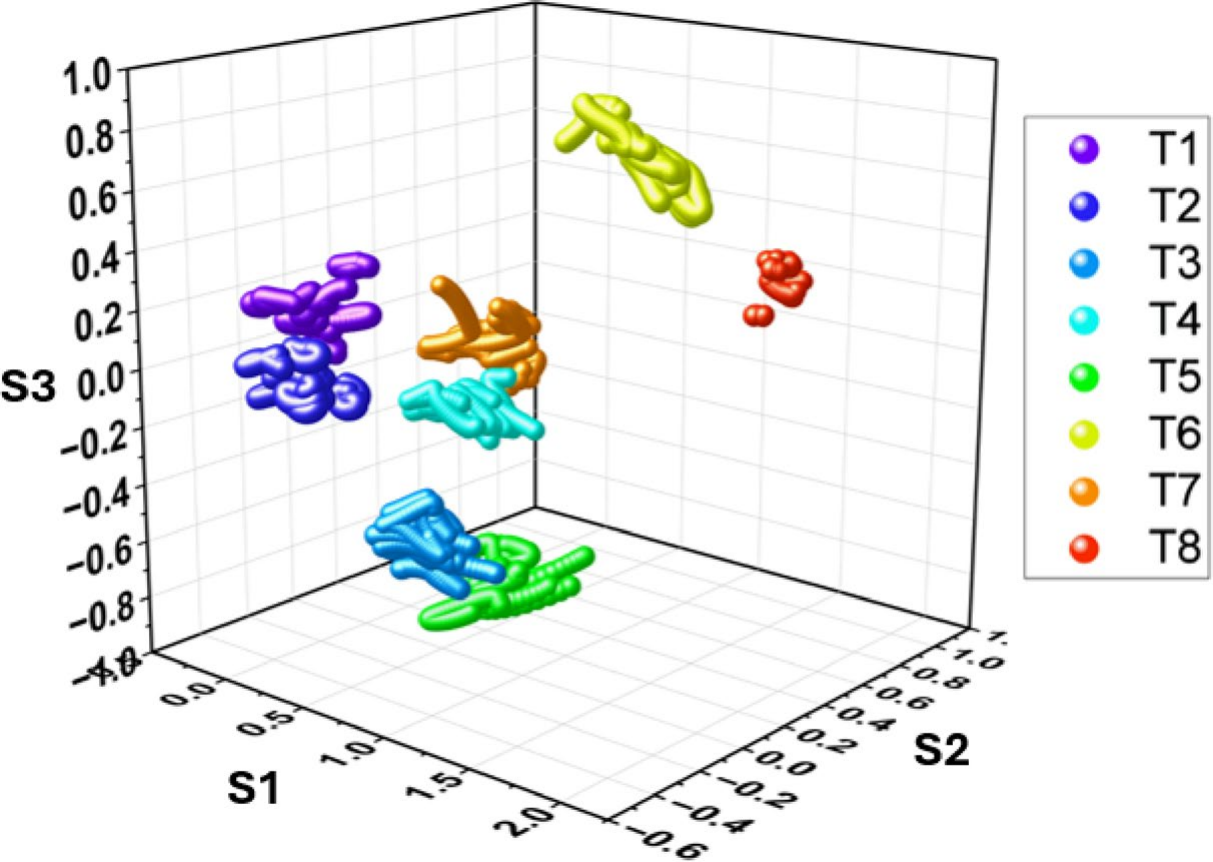
Representation of task in the latent space defined by the first 3 synergies. The central 0.5 s of the task performed by a randomly selected participant were projected onto the synergy space to visualize movement separability. The image was plotted using the software OriginPro 2024 (Learning Edition), https://www.originlab.com.

The data from all 8 tasks were projected to only 3 synergies, which explained the variance of > 80%. The data of projected postures for a randomly selected participant is shown in Fig. 13. Such a representation for all participants is provided in the supplementary material (Fig. S2). Visually, the tasks seem well represented in the latent space spread in distinct areas. However, to quantify the separability, a classification based on logistic regression was performed. The classification algorithm was trained over 60% of the training data, and the classification results for 40% of the test data were computed for each participant.

**Fig. 14:**
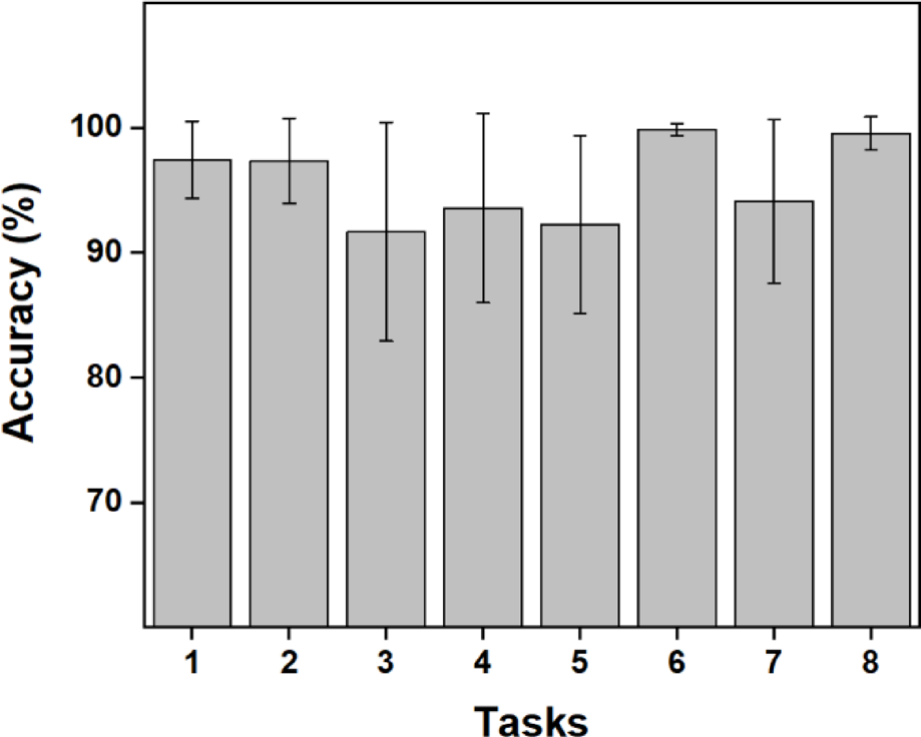
Average accuracy of the classification performed to quantify movement separability in the latent space. The accuracy represented by the F-1 score was averaged across participants for each task separately. The error bars indicate S.D.

The accuracy represented by the F-1 score was averaged for each task across all participants. And presented in Fig. 14. The results showed that the classification algorithm could classify the task with an average accuracy of at least 90%. The lowest classification accuracy was observed for tasks 3 and 5, which lie very close in the latent space, also visible in Fig. 13. A higher classification score across the tasks in the latent space indicates better separability of tasks in the latent space suggesting that the bimanual postural information is still retained in the latent space.

## Discussion

Previously, studies on human hand kinematics aimed to develop a simplified lower-dimensional representation of the human hand by exploring synergies. This has been explored mainly for the dominant hand to simplify control of higher dimensional systems like the prosthetic hand. In this paper, an attempt was made to expand on this work by evaluating if a single set of bimanual synergies could control the movements of both hands. While an initial idea on bimanual synergies for hand movements was presented previously^14^, and its application demonstrated for proximal arm movements in^11^, in this study, a thorough analysis of the synergies, posture reconstruction, and classification of tasks from latent space, was performed. The participants performed only asymmetric finger movements involving eight tasks.The result of the current study indicates that the first three synergies account for > 80% variance in data for the movements selected. This shows that very few synergies can control the movement of fingers of both hands. Such high variance for lower-order synergies has been previously observed for single-hand kinematics involving fewer joints^6,8,10^. Still, the current results indicate that the CNS uses simplified control units that control the movement of both hands. Additionally, the results show that the top 5 synergies explain greater than 90% variance in data. Thus, low dimensional control would suffice for controlling the movements of a prosthetic hand for bimanual movements. We also presented the synergies encoded by the DO and NDO hands. The first synergy encoded symmetric movements, while the remaining synergies encoded asymmetric movements, which were dependent on the tasks selected. The first three synergies were similar across participants, whereas the higher-order synergies were different, indicating the involvement of participant-specific strategies for task performance. Further analysis of reconstruction error showed that the tasks could be reconstructed using just 3 synergies that yielded an RMSE of 4°. Adding more synergies improved performance; however, adding more synergies in the NDO hand would induce oscillations that are artifacts picked up by the NDO synergies from the DO hands. A tradeoff between accuracy and oscillations is necessary while selecting the number of synergies for reconstruction. While the errors seem to be high for some of the plots shown, it should be noted that the plot shown is for the participant who demonstrated the maximum error. Finally, the classification algorithm showed an average accuracy of 90% for all tasks. This further supports the hypothesis that the bimanual synergies could be used to control the movements of the DO and NDO hands since the task information is still retained in the lower dimensional subspace.

Thus, the findings from our study suggest that a small set of synergies can control bimanual finger movements and complements our traditional understanding of brain lateralization. Brain lateralization refers to the specialization of the two hemispheres, where each hemisphere is responsible for different functions or tasks. Typically, the left hemisphere controls fine motor skills in the right hand, and the right hemisphere handles visuospatial processing and motor tasks involving stability for the left hand. This concept suggests that the control of movements in each hand would require independent neural mechanisms, reflecting the distinct roles of each hemisphere. However, the study’s results, supported by a preliminary study^14^, indicate that the central nervous system (CNS) might also use a coordinated and a simplified control strategy for both hands. The fact that the first three synergies account for over 80% of the variance in bimanual movements implies that both hands can be controlled by similar neural mechanisms, thus suggesting that the function played by each hand can be controlled by different lateralized mechanisms that nonetheless need synchronization. This is particularly significant because it hints at a more integrated approach by the CNS in handling bimanual coordination, where control units (synergies) are shared across both hemispheres. In addition to the lateralization model, which emphasizes hemispheric specialization and division of tasks, the study’s findings support the idea of a more symmetrical and coordinated control strategy for well-practiced tasks. If a small set of synergies can manage movements in both hands, it suggests that motor control may be less compartmentalized between hemispheres and more globally coordinated.

The obtained synergies probably arise from the synchronization of cortical and subcortical structures. For instance, when one hand holds an object while the other manipulates it, the CNS must synchronize excitation and inhibition across hemispheres. Such synchronization could happen through cortical mechanisms facilitated by callosal connections, which may inhibit the opposite hemisphere to prevent interference between the two hands. To support this, previous studies have shown that interhemispheric inhibition (IHI) plays a crucial role in bimanual coordination, with the corpus callosum acting as a key mediator of inhibitory and excitatory signals between hemispheres^31,32^. For example, holding an object steady with one hand may involve the excitation of motor areas in one hemisphere while simultaneously inhibiting the corresponding areas in the opposite hemisphere to prevent unwanted movements^33,34^. Additionally, subcortical structures, such as the basal ganglia and cerebellum, may also play a role, particularly in highly practiced tasks, by fine-tuning motor commands and coordinating movements between the hands^35,36^. These structures contribute to the timing and sequencing of bimanual movements, particularly in tasks requiring precise temporal coordination as performed in the current study^37,38^. Thus, as demonstrated in this study, the coordination of bimanual movements in sequential tasks may rely on interhemispheric communication through the corpus callosum and other subcortical pathways, which facilitate the coordination of timing and force between the hands, enabling smooth and efficient task performance. The similarities observed in the first three synergies among participants indicate that these neural mechanisms are consistent across individuals, highlighting an important characteristic of bimanual motor control.

Finally, previous studies have also shown that task demands play a significant role in determining the extent of lateralization, influencing how the brain allocates resources across the hemispheres for motor control and coordination. As tasks become more complex, the degree of lateralization tends to shift towards a more focused lateralized control, revealing a dynamic relationship between task demands and brain specialization. The role of interhemispheric collaboration as task demands increase has been previously shown through several studies involving visual field stimuli experiments^39,40^, EEG measurements^41^, the role of prior task knowledge on hemispheric contributions^28^, and changes in prehensile synergies based on task demands^42^. In our current study, regular tasks involving manipulations of everyday objects were utilized, thus allowing a simplified control of both the dominant and non-dominant hand involving interhemispheric and subcortical involvement. How would the synergies differ as task demands increase must be studied further.

One of the main limitations of the current study is the number of tasks performed by the participants. It is not unreasonable to expect that the observed results have been influenced by a limited number of tasks employed in the study. Considering existing literature, some of the commonly used tasks in the analysis of synergies involve grasping objects^6,10,16,21,43,44^, activities of daily living—single hand^22,45^, and Cooking tasks- Bimanual^46^. Tasks involving grasping objects will result in a joint angle movement pattern, as shown in Fig S3(a) in the supplementary material. These tasks usually involve a reach phase, a grasp phase, and a drop phase. Thus, the joint angles vary from a steady posture to a final static posture. The angle then returns back to the initial condition. Such a variation is also observed in datasets that use activities of daily living like reaching for a brush and then holding the brush and brushing, holding a comb and combing, and using a knife to cut vegetables^22,45^. In these tasks, upon holding an object like a comb or a knife, the joints of the fingers are locked, whereas proximal joints perform the movements. Finally, only one dataset involved bimanual tasks in cooking tasks. Unfortunately, even this dataset used tasks that involved using a spoon to beat eggs and moving a jar from one place to another. Only a few tasks involved opening the lid of a bottle and opening the covering of a bread packet that would result in a joint angle pattern, as shown in Fig. S3(b) (check supplementary material), which is desirable since it involves continuous manipulation mainly using the distal joints. Hence, only those limited tasks that require significant variations in joint angles of the fingers, as shown in Fig. S3(b), were selected for the current study as opposed to those tasks that require the fingers to be locked, and task performance is based only on proximal segments. Secondly, it should be noted that the derived bimanual synergies are a mix of dynamic and postural synergies. This is because the tasks were selected as per the dynamic dominance hypothesis, which involved static postures of the NDO hand and dynamic movements of the DO hand. Even though dynamic and simultaneous dissimilar movements of both DO and NDO hands are rarely performed in everyday tasks, further experiments are necessary to consider the effect of such movements on the derived synergies. Finally, the first 4 tasks are all rotation tasks involving changes in the finger aperture. While the relation among joint angles remains consistent, there could be differences in kinematic synergies due to subtle adjustments in hand positioning, grip adaptation, and the interaction between the two hands due to differences in the object sizes. Such tasks were greater in number since rotation tasks provide maximum involvement of distal segments without any involvement of the proximal segments.

As a future scope, further analysis must be performed to assess the influence of task complexity. In the current study, the experiment protocol also included a second task wherein, the participants performed the same task but with the roles reversed. In other words, the DO hand held the object steady while the NDO hand performed movements like opening the lid, etc. How the synergies differ in this case will be studied further as part of the future scope of this paper. Additionally, an analysis of handedness could be performed using the dataset. Based on the dynamic dominance hypothesis, the DO hand is optimized for smooth movements, while the NDO hand is tuned to hold the object steady^47,48^. Since the second task was opposite to the expected nature of the hand movements, how the synergies differ in response to the inherent handedness-related nuances could be studied. Finally, in the current study, some of the tasks also involved proximal segment movements. Since the proximal segment movements were not measured, further studies involving the influence of these movements on distal segment synergies must be explored.

## Concluding comments

In conclusion, this study demonstrates that a small set of synergies can effectively control bimanual finger movements. The results show that the first three synergies account for over 80% of the variance, suggesting that both hands can also be governed by unified control strategies rather than independent neural mechanisms across hemispheres. This involves interhemispheric interaction and sophisticated coordination between the cortical and subcortical structures, especially when the tasks are well practiced, such as daily life activities. This integrated control approach highlights the potential of using low-dimensional synergies for prosthetic applications, supporting simplified motor control even in bimanual tasks. However, the extent to which these synergies adapt to increased task complexity and handedness-related nuances remains an open question. Future work will explore the impact of more intricate tasks, role-reversal scenarios between hands, and handedness dynamics to further understand how synergies evolve and how interhemispheric coordination shapes motor control.

## Electronic supplementary material

Below is the link to the electronic supplementary material.

Supplementary Material 1

## Data Availability

The data collected for this study is available upon request by contact with the corresponding author.
