# Supplementary material for: Exploring synergistic patterns in bimanual distal limb movements through low dimensional representations
Source: Sci Rep. 2025 May 23;15:17943. doi: 10.1038/s41598-025-02680-x (PMC12102304; doi:10.1038/s41598-025-02680-x)
Supplement: Supplementary file 1 — Supplementary Material 1 [file 41598_2025_2680_MOESM1_ESM.docx]

1. **Table S1: Details of various tasks performed in the study**

| **Sl. No.** | **Object used for manipulation** | **Task performed** | **Complementary movements in adjacent trials?** |
| --- | --- | --- | --- |
| 1. | Pen | Lift the pen from the specified area on the table with the NDO hand, hold the top grip section barrel with the NDO hand, and rotate the bottom barrel thrice in the CW direction and thrice in the CCW direction using the DO hand. The NDO hand keeps the pen in the specified region on the table. | No |
| 2. | Screw and nut | Lift the screw holding its head with the NDO hand. Use finger movements of the DO hand to rotate the nut thrice in the CW direction, then thrice in the CCW direction. The NDO hand is used to keep the screw back on the table | **No** |
| 3. | Pencil box with a screw cap | Lift the pencil box with the NDO hand. Use finger movements of the DO hand to rotate the cap thrice in the CW direction, then thrice in the CCW direction. The NDO hand is used to keep the box back on the table | **No** |
| 4. | Hair gel box | Lift the hair gel box with the NDO hand. Use finger movements of the DO hand to rotate the lid thrice in the CW direction, then thrice in the CCW direction. The NDO hand is used to keep the box back on the table | **No** |
| 5. | Pull open the lid and unscrew/screw the top cover of a bottle | Lift the bottle with the NDO hand. Use finger movements of the DO hand to pull open the lid, place it on the table, then unscrew the cover by rotating thrice in the CCW direction using the DO hand. The object is placed back on the table using the NDO hand. This is done for the 1^st^, 3^rd^, 5^th^, 7^th^ and 9^th^ trial.  For the 2^nd^, 4^th^,6^th^, 8^th^, and 10th trials, the NDO hand is used to lift the object and tighten the cover by rotating in the CW direction thrice using the DO hand. Lift the lid and push-fit using the DO hand. The NDO hand is used to place the bottle back on the table | **Yes** |
| 6. | Manipulating a pipe wrench | Lift the pipe wrench with the NDO hand. Use the index and thumb of the DO hand to rotate thrice in the CW and thrice in the CCW direction. The NDO hand is used to keep the wrench back on the table. Anti-phasic movements are performed by the index and the thumb of the DO hand while manipulating. | **No** |
| 7. | Placing and removing objects from a box | The box (initially with the lid closed) is lifted from the table using the NDO hand. The DO hand is used to open the lid, lift the object from inside the box and place it on the table. The NDO hand is used to place the box on the table. This is done for the 1^st^, 3^rd^, 5^th^, 7^th^ and 9^th^ trial.  For the 2^nd^, 4^th^,6^th^, 8^th^, and 10th trials, the NDO hand is used to lift the box (with the lid in open condition). The DO hand is used to lift the object from the table, keep it inside the box, and close the lid. The NDO hand keeps the box back on the table. | **Yes** |
| 8. | Dropping coins in a piggy bank | Lift the piggy bank from the specified area on the table with the NDO hand. The DO hand is used to pick coins and drop them in the piggy bank. | **No** |

1. **Principal Component analysis to obtain Bimanual synergies**

- Each trial's data is 800 (8 sec x 100 samples/sec) x 128 (16 sensors x 4 valued quaternions x 2 hands). Relative quaternions are computed for each trial using Eq (2), thus obtaining quaternions that represent the orientation of each joint. Since there are 15 joints per hand, each trial data is of size 800 x120 (15 joints x 4 valued quaternions x 2 hands).
- For each participant, the data of all trials of all tasks are concatenated vertically. Thus, the data matrix is of the size 64000 (800 samples /trial x 10 trials x 8 tasks) x 120.
- The mean of quaternions of each joint is computed using Markley’s algorithm ^29^ . Since direct averaging is not applicable to quaternions, Markley demonstrated that the eigenvector corresponding to the maximum eigenvalue of a quaternion array represents the mean of the array.
- The mean is subtracted from each column using the conjugate quaternion operation (Eq 2).
- For linearizing a quaternion, the quaternion of the from q= (q_w_, q_v_) was mapped to a 3D plane using the logarithmic mapping (Eq 3). The new element in the 3D plane will be a 3D tuple v= [$v_{x}, v_{y}, v_{z}]. This$element v encodes information of both axis and the corresponding angle. The MATLAB function ‘quatlog’ was used for the calculation.

$v=ln q= \left( ln(\left| q \right|, \left( \frac{1}{\left\| q_{v} \right\|} arcos \frac{q_{w}}{\left| q \right|} \right)q_{v} \right)$ (3)

After logarithmic mapping, each 4 valued quaternion is converted to 3 valued linearized vectors. Thus, the size of the linearized data matrix is 64000 x 90 (15 joints x 3 valued vectors x 2 hands).

- PCA using eigenvalue decomposition is applied to the linearized data matrix to obtain a set of eigenvalues (90 x1) and eigenvectors (90 x 90).
- The eigenvectors (synergies) are sorted in decreasing order of their corresponding eigenvalues.
- Top k synergies (90 x k) that explain at least 90% variance in data are selected for analysis.
- Eigen postures are plotted by rotating the synergies around the mean posture using the quaternion multiplication operation (Eq 4 and 5).

${Eigen posture}_{max}={qs}_{i} \otimes{q\mu}_{i}$ (4)

${Eigen posture}_{min}={{qs}_{i}}^{conj} \otimes{q\mu}_{i}$ (5)

Here, ${qs}_{i}$ is the ith synergy, and ${q\mu}_{i}$ is the mean posture.

- To reconstruct the original data from the reduced dimensions, the linearized data was projected onto the reduced dimensional space, and then the linearized data was converted back to the original quaternion using exponential mapping (Eq 6). The MATLAB function ‘quatexp’ was used for the calculation.

$q=e^{v}$=$\left[ cos\left( \frac{\theta}{2} \right), sin\left( \frac{\theta}{2} \right)\frac{v}{\left\| v \right\|} \right]$ (6)

where $\theta=\left\| v \right\|$

- Mean was added to the reconstructed quaternion using $q_{mean}\bigotimes q_{i}$. Here $q_{i}$ represents individual reconstructed quaternions of that column.
- Reconstruction error was computed using Eq (7) as suggested in ^27^.

$RMSE= \sqrt{\sum_{i=1}^{n} \frac{1}{n}\left\| \ln({Q_{1}}^{conj}\bigotimes Q_{2} ) \right\|^{2}}$ (7)

1. **FIG S2: Plots depicting the separability in tasks when projected into the top 3 synergies. The plots are shown for all participants P1-P16.**


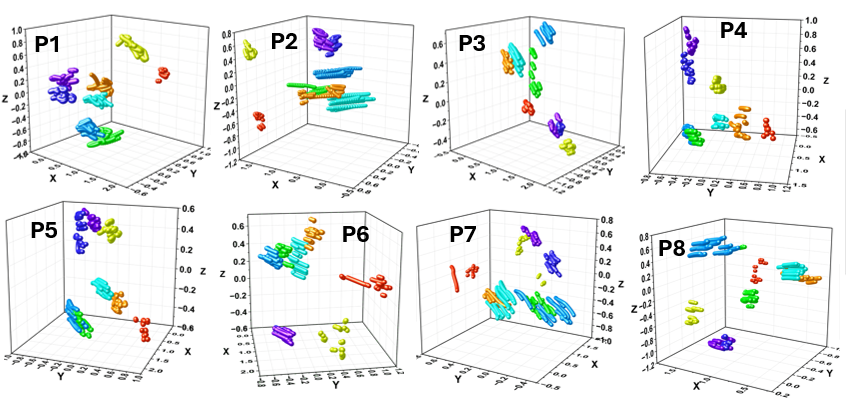


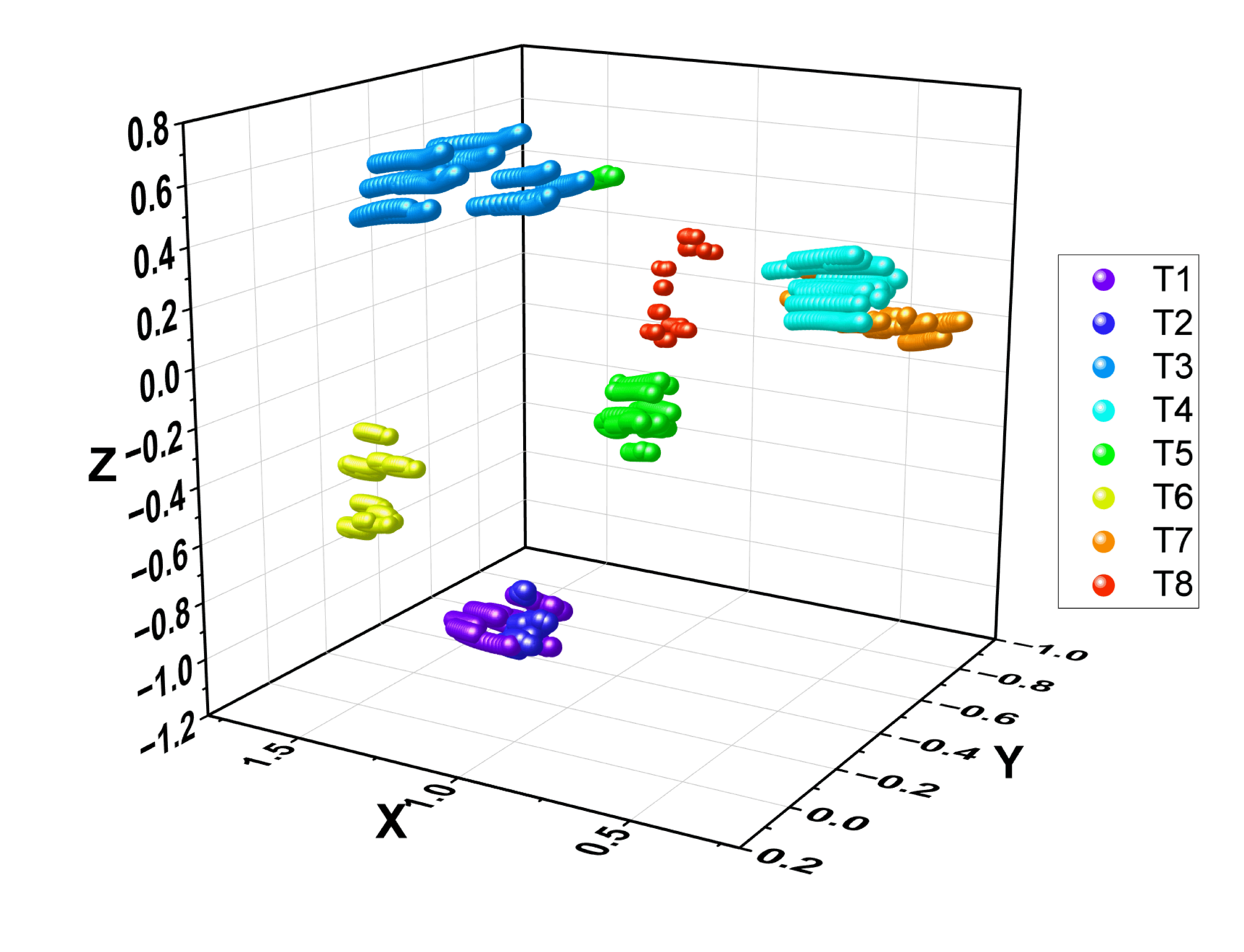


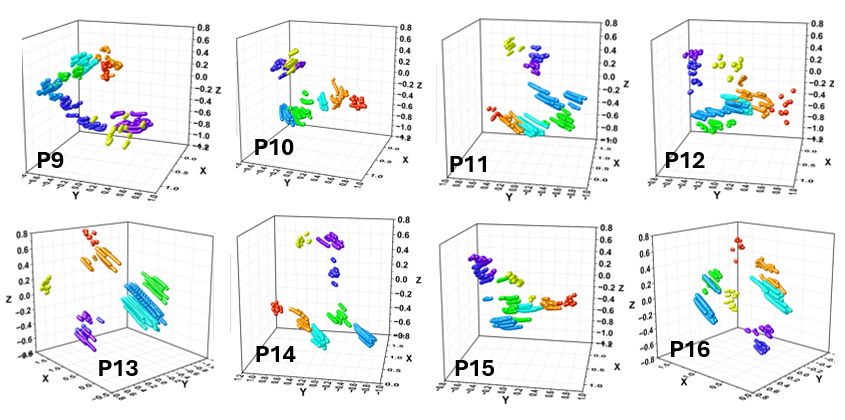


1. **FIG S3: Plots depicting the representative variation in finger joint angles. Figure (a) represents regular tasks, such as picking an object or grasping a handle**. **Figure (b) represents object manipulation tasks like opening the lid of a bottle**


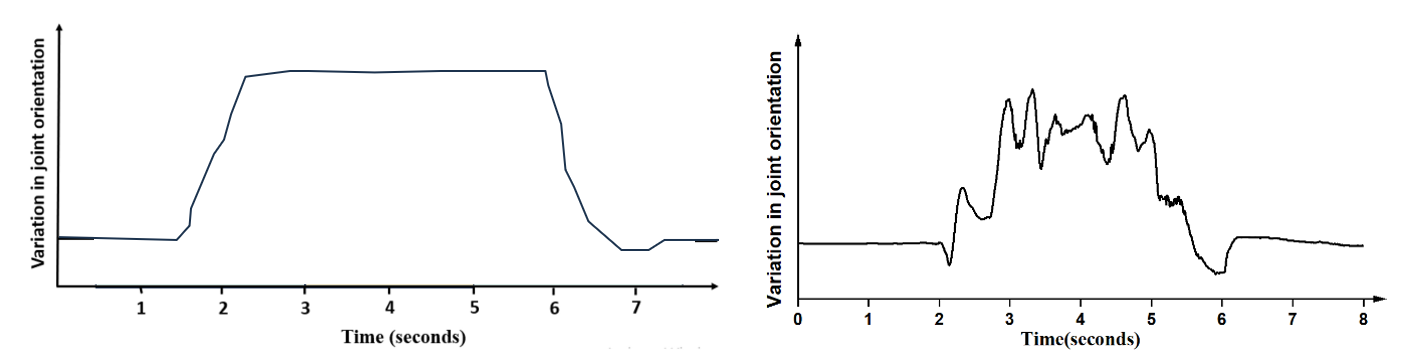


**(a)**


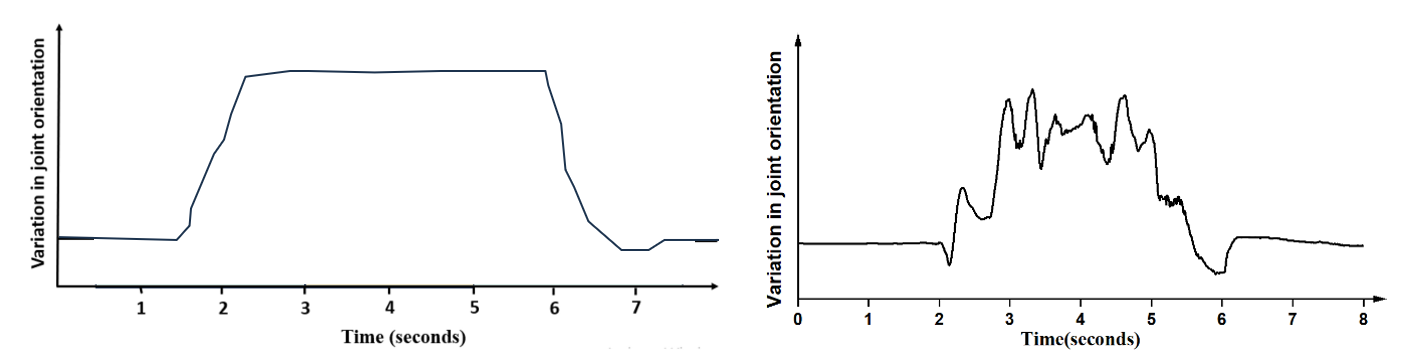


**(b)**
